# Clustering of trauma patients based on longitudinal data and the application of machine learning to predict recovery

**DOI:** 10.1038/s41598-022-21390-2

**Published:** 2022-10-10

**Authors:** Kostas Stoitsas, Saurabh Bahulikar, Leonie de Munter, Mariska A. C. de Jongh, Maria A. C. Jansen, Merel M. Jung, Marijn van Wingerden, Katrijn Van Deun

**Affiliations:** 1 Department of Methodology and Statistics, Tilburg University, Tilburg, 5000 LE The Netherlands; 2 Department of Cognitive Science and Artificial Intelligence, Tilburg University, Tilburg, 5000 LE The Netherlands; 3 Department Traumatology, ETZ Hospital, Hilvarenbeekseweg 60, 5022 GC Tilburg, The Netherlands; 4 Network Emergency Care Brabant, Brabant Trauma Registry, Hilvarenbeekseweg 60, 5022 GC Tilburg, The Netherlands

**Keywords:** Prognosis, Quality of life, Health care, Rehabilitation

## Abstract

Predicting recovery after trauma is important to provide patients a perspective on their estimated future health, to engage in shared decision making and target interventions to relevant patient groups. In the present study, several unsupervised techniques are employed to cluster patients based on longitudinal recovery profiles. Subsequently, these data-driven clusters were assessed on clinical validity by experts and used as targets in supervised machine learning models. We present a formalised analysis of the obtained clusters that incorporates evaluation of (i) statistical and machine learning metrics, (ii) clusters clinical validity with descriptive statistics and medical expertise. Clusters quality assessment revealed that clusters obtained through a Bayesian method (High Dimensional Supervised Classification and Clustering) and a Deep Gaussian Mixture model, in combination with oversampling and a Random Forest for supervised learning of the cluster assignments provided among the most clinically sensible partitioning of patients. Other methods that obtained higher classification accuracy suffered from cluster solutions with large majority classes or clinically less sensible classes. Models that used just physical or a mix of physical and psychological outcomes proved to be among the most sensible, suggesting that clustering on psychological outcomes alone yields recovery profiles that do not conform to known risk factors.

## Introduction

Prediction of the course of disease is an important aspect of personalized medicine^1^. Variability among patients, in etiology and in treatment often causes inadequate prognostic estimates for a single predictor^2^. Therefore, clinical and patient characteristics are often combined in a multiple predictor model.

Outcome prediction for a heterogeneous disease such as injury (physical trauma) is complex; it affects all levels of society, covers multiple body regions, includes many mechanisms and causes, and could range from minor severity to complex poly-trauma. However, Injuries are responsible for an estimated 6% of all years lived with disability and therefore a major public health problem^3^. In 2019, nearly 80,000 people (incidence: 45 per 10,000 persons) were admitted to a hospital due to injuries in the Netherlands of which 97% survived^4^. Data-driven prognostic models could be used to inform patients and clinicians about their predicted recovery, target patients that are at high-risk for poor recovery and improve shared decision making between patient and clinician^5,6^.

Prognostic models in trauma care have a long history; survival prediction models are already embedded for trauma care quality assessment^7–10^. However, it is widely acknowledged that prediction of survival alone is insufficient and should be extended to recovery after non-fatal outcome^11^. Recovery after injury is not only based on physical functioning, but also includes emotional aspects^12^. This holistic focus on recovery after injury is substantiated by the findings of previous research, i.e. injury survivors often experience symptoms of post-traumatic stress, anxiety and depression, in addition to poor health related quality of life^13^.

A number of prognostic models have been developed to predict functional outcome after injury^14–17^ and psychological outcome after trauma^18–20^. Although previous literature recognized the interchangeable effect of psychological outcome and functional outcome^21^, both outcomes are not yet incorporated in one prognostic model. Furthermore, the models are mostly developed using traditional regression techniques. Currently, more sophisticated and advanced mathematical machine learning (ML) techniques are available with potential in trauma care^22,23^. ML has the ability to deal with the heterogeneity of the trauma population and the multidimensional burden by including multiple outcome measures, nonlinear associations and interactions in a model comparison approach. However, non-linear or ensemble models do not always provide beneficial performance over logistic regression and model complexity should be weighed against model transparency^23,24^.

In the current study, clustering of patients who suffered an injury is performed based on longitudinal recovery profiles (physical and psychological). Obtained clusters are evaluated based on statistical parameters and medical expertise in order to end up with clusters that are clinically sensible and recapitulate clear separation across known risk factors for trauma recovery^25^. Several machine learning models were trained and evaluated for the prediction of trauma groups. The aim of the study is to develop machine learning models which will be characterized not only by high prognostic performance but also from high medical validity. We demonstrate that ML can be used for predicting mid and long term physical and psychological outcome after injury. To our knowledge, no previous study has used ML for investigating these combination of outcome domains after injury. Short-term outcomes, such as mortality and complications, are most prevalent outcomes of interest. More particularly, for prediction of death after trauma an accuracy of 97% has been reported based on ML methods with demographics and clinical variables as predictors^26^. Additionally, accurate ML models (accuracy higher than 70%) applying routinely clinical data have been developed to predict complications such as acute respiratory distress syndrome, multi-organ failure and Venous thromboembolism after trauma^27,28^.

## Methods

### General methodology

During this study longitudinal data that relate with the recovery of patients were used to cluster patients. More specifically, we considered four different variations of longitudinal data with three unsupervised clustering methods. Longitudinal data which represent physical or psychological recovery and a combination of both were considered. Additionally, one more case of physical recovery with pre-injury values was applied. Principal Component Analysis (PCA) was executed to examine the correlation of physical and psychological longitudinal data and confirm that psychological or a combination of psychological and physical longitudinal data can be employed for unsupervised clustering. The next step was to determine the optimum number of clusters based on different statistical criteria such as Gap Statistic and Bayesian Information Criterion (BIC). Application of different machine learning algorithms together with over-sampling and under-sampling for the prediction of the generated clusters followed. As predictors for the machine learning models were demographic data and clinical injury-related data from the patients. Parallel with machine learning part, qualitative evaluation of the generated clusters occurred based on descriptive statistics and medical expertise in order to assess how clinically sensible the generated clusters were. Multivariate Analysis (MANOVA) was used as supplementary method for the evaluation of the clusters. Aim of the present study was to develop models with high predictive performance and high clinical validity (see Fig. S1 in Supplementary information for a schematic overview of the study).

### Dataset

#### General

In this study, the Brabant Injury Outcome Surveillance (BIOS) dataset was used. BIOS is a longitudinal, multi-center follow-up study among all admitted adult injury patients in the trauma region Noord-Brabant (The Netherlands)^29^. Patients were eligible if they were admitted to an Intensive Care Unit (ICU) or ward within 48 h after injury and survived hospital discharge between August 2015 and December 2016. Exclusion criteria were pathological fractures (i.e. malignancy), no permanent address and if they were unable to answer the Dutch follow-up questionnaires. Out of in total 9774 patients admitted to trauma care in this period, 4883 patients participated in the BIOS study. Informed consent was obtained from patients or their proxies. The study was approved by the Medical Ethics Committee Brabant (no. NL50258.028.14) and all methods were performed in accordance with the relevant guidelines and regulations. The dataset was anonymized prior to analyses.

#### Longitudinal data for clustering

In the BIOS dataset, the patient reported outcome measures were recorded for different time points i.e. one week (*T*_1_), one month (*T*_2_), three months (*T*_3_), six months (*T*_4_), twelve months (*T*_5_) and twenty-four months (*T*_6_) after injury. There are two primary categories of these outcome measures: physical health related items and psychological health related items. In the physical health domain, the EuroQoL-5D, with EQ-5D utility (‘EQ-5D’) and EQ-Visual analogue scale (‘EQ-VAS’)^30^ along with the Health Utilities Index (‘HUI2’ and ‘HUI3’)^31^ were used to measure health. Pre-injury values for the variables EQ-5D and EQ-VAS namely ‘Pre-injury EQ-5D’ and ‘Pre-injury EQ-VAS’ were also recorded. In the psychological domain, the Hospital Anxiety and Depression Scale (HADS) was used to screen for and record symptoms of anxiety and depression (‘HDSA’ and ‘HDSD’ respectively)^32^. Additionally, The Impact of Events Scale (‘IES’) was used to measure Post Traumatic Stress Symptoms (PTSS)^33^.

#### Predictors for Machine Learning modelling

For the development of models, which will predict the trajectory of recovery (physical, psychological and combination), two categories of prognostic factors were chosen namely demographic and clinical-injury related variables. The selection of the variables was based on clinical experience and previous literature^34–37^. In total twenty-six predictors are considered. Nine of them are numerical while the rest seventeen are categorical. For the categorical variables we apply the function *get_dummies* from pandas in order to convert them to dummy/indicator variables.

From the demographic data, variables such as ‘Gender’, ‘Age’, Body Mass Index (‘BMI’), ‘Status score’, pre-injury health (‘Pre-injury EQ-VAS’, ‘Pre-injury cognition’), ‘Frailty’ and ‘Education level’ were considered. ‘Status score’ represents social economic status and was based on home postal codes in the Netherlands. These values were based on the level of education, income and percentage of unemployment in the neighborhood of patients’ permanent residence. ‘Frailty’ was measured at one week or one month after injury with the Groningen Frailty Index and refers to the condition before the injury^38^. ‘Education level’ was categorised in three levels: low (primary education, preparatory secondary vocational education or without diploma), middle (university preparatory education, senior general secondary education or senior secondary vocational education and training), and high (academic degree or university of applied science).

Clinical injury-related data of the included patients were collected from their electronic medical files and included the Abbreviated Injury Scale (AIS). The AIS classifies all injuries in a specific code, including the severity, location and type of injury. In total, twelve injury predictors were created: three lower extremity injury groups (pelvic injury, hip fracture, and tibia fracture/complex foot or distal/shaft femur fracture), two upper extremity injury groups (shoulder and upper arm injury, and radius, ulna or hand fracture), one group of Traumatic Brain Injury (TBI), one face injury group, two thorax injury groups (thorax injury and rib fracture), one abdomen injury group and two spine injury groups (spinal cord injury/brachial plexus lesion and stable vertebral fracture/disc injury). The AIS severity score of the three most severely injured body regions are squared and summed to provide the ‘Injury severity score’ (ISS)^39^. Low values of ‘Injury severity score’ indicate minor injury while higher values indicate a serious one. AIS codes for body regions were used to classify patients recovery. Additional injury-related collected predictors are length of hospital stay (‘Admission days in hospital’), ‘Type of accident’ (sports, violence, home, traffic etc.) and comorbidity which is measured with two variables. The one variable is ‘Comorbidities’ which is the absolute values of comorbidities and the other is ‘Type Comorbidity’ measured with the American Society of Anesthesiologists (ASA) physical status classification system ranging from zero (healthy patient) to four (severe systemic disease that is a constant threat to life). An overview of the longitudinal variables used for clustering and the variables used as predictors can be found in the table S2 of Supplementary information.

### Treatment of missing values

Upon initial analysis of the dataset, it was observed that there are several missing values. The BIOS dataset, as many longitudinal datasets, suffers from a sizeable number (39.3%) of missing values. In order to deal with the incomplete data, imputation of the missing values was implemented. The imputation was done using the R package "MICE". Missing data that occurs in more than one variable in a dataset generates a challenge. Multivariate Imputation by using Chained Equations (MICE) is a method for complex incomplete data problems^40^. MICE can handle multivariate imputation by relying on a set of conditional densities for each variable. It starts from an initial imputation and draws the subsequent imputations by iterating over the conditional densities.

### Principal component analysis

For the exploration of the General health of the patients a combination of Physical and Psychological variables was used. In order to investigate the correlations between the variables, a Principal Component Analysis was carried out to summarize the variable scores by a few components and visualize the variation and covariation between variables. Before executing the PCA, Psychological variables were multiplied with minus one to align their severity axis with variables that represent Physical Health, as low values of Psychological Health indicate low depression and anxiety (good General Health) while low values for variables that represent Physical Health suggest poor General Health (reverse coding). Transformation of Psychological variables occurs only for PCA. Additionally, since variables are measured on different scales we decided to standardize the scores to z scores before feeding the data for PCA (with the z score obtained by centering and scaling to unit variance). We applied the function *prcomp* which is a default function from the R base package for PCA. The graph of the PCA results was made using the "factoextra" R package.

### Clustering

Longitudinal data are ubiquitous in medical research. Making individual point predictions for a number of outcome variables could be hard to weigh and interpret in the shared discussion making process. Here, to facilitate these discussions, we sought to group longitudinal profiles into clusters of similar outcome trajectories that represent clinically clearly defined outcomes. We compared several methods for clustering longitudinal data: kml3d, HDclassif and Deepgmm. kml3d is a popular method to cluster multiple trajectories in medical research. kml3d is a variation of kml, a method based on k-means for cluster modelling of longitudinal data^41,42^. Traditionally, the k-means clustering method uses an iterative algorithm consisting of two phases. In the first phase, k points are randomly initialized as cluster centers in the data space. A partition is then defined from these points by attributing each data point to the nearest cluster center according to a predefined distance criterion. The partitions are then updated and their cluster mean is recalculated, replacing the cluster center. This step is then repeated until convergence of the cluster centers. For the longitudinal kml3d method the function is essentially identical. The k points are replaced with randomly sampled sequences, and the distance is calculated through the panel of existing distance for longitudinal data. The kml3d method clusters several variable trajectories jointly. This means that multiple continuous correlated variables are summarized into a single nominal cluster variable that contains the information of the correlated variables. HDclassif^43^ is a Bayesian clustering technique based on the assumption that data with high dimensionality can be adequately described in low dimensional sub-spaces, proposing a new parameterization of the Gaussian mixture model that combines the ideas of dimensionality reduction and constraints on the model. Deepgmm^44^, Deep Gaussian Mixture Modeling, is an extension of classical Gaussian mixtures to multiple layers. Layers contain a set of latent variables that follow a mixture of Gaussian distributions. To prevent solutions with an excessive number of parameters, dimensionality reduction is applied at each layer by way of factor models.

After imputation of the missing values, but before feeding the variables to the clustering methods, continuous variables were normalized to z-scores, as the variables in the dataset were measured on different scale (e.g. ‘EQ-VAS’ 0–100, ‘EQ-5D’ 0–1).

### Determining the optimum number of clusters

Besides the visual Elbow method, the Kml3d library offers different criteria (Calinski, Ray, Davies) in order to determine the optimum number of clusters. However, for the BIOS data, contradictory and non-conclusive results were obtained applying the kml3d criteria. For this reason, the optimum number of clusters was calculated using the gap statistic obtained from the "Nbclust" library in R^45^. The cluster analysis selector for "Nbclust" was set as k-means since kml3d method is also based on k-means.

In the case of HDclassif clustering method, a grid search on the different parameters of the method was performed to discover the optimum number of clusters. We set four different parameters in order to obtain the cluster representation with the highest BIC value. The first parameter is the number of clusters which varies from 3 to 10. The second parameter is the applied algorithm which can be in three forms: (1) The standard expectation–maximization algorithm namely EM, (2) The classification expectation–maximization algorithm namely CEM and (3) The stochastic expectation–maximization algorithm namely SEM. The third parameter is the method of initialization. HDclassif provides three different initialization methods: k-means, random and param (initialization with the means being generated by a multivariate normal distribution and the covariance matrix being common to the whole sample). Finally, HDclassif makes use of twelve models with class specific covariance matrices and two models with a common covariance matrix.

A similar approach as with HDclassif was adopted for the calculation of the optimum number of clusters obtained with the Deepgmm method. The expectation maximization algorithm used by Deepgmm also requires initialization. In this case, the initialization is done by partitioning the dataset and then estimating the initial values for model parameters based on these partitions. There are three options available in Deepgmm for the initial partition of the data: random partitioning, clustering using the k-means algorithm of Hartigan-Wong and agglomerative hierarchical clustering^44^. The number of hidden layers can vary from one to three. For the scope of this study, the number of hidden layers was limited to one. For all possible combinations of initialization and number of hidden layers the BIC was calculated.

### Prediction modelling

For the supervised modelling part, the cluster label corresponding to the multivariate recovery trajectory of the patients is set as their class membership and used as a target for prediction. Logistic regression, Random Forest and XGBoost were applied for predicting the class a patient belongs to using demographic data as predictors. Note that the classes were not pre-existing but derived from the clustering step that only considered outcome variables. The optimal setting of the different algorithms occurs by splitting the data in a random fashion. 80% of the data was used for training and tuning the hyper parameters. The remaining 20% of the data was used for evaluation of the optimized algorithm. Stratified cross validation with five folds was applied to calculate the 95% confidence interval (CI) of the accuracy. All model classes were subjected to hyperparameter optimization (see table S3 of Supplementary information) for the maximization of the objective function which is accuracy (or minimize the classification error). Since we deal with unbalanced data it was decided to perform under-sampling and over-sampling (smote or traditional over-sampling) using the "imblearn" library in Python. Undersampling and oversampling are techniques used to combat the issue of unbalanced classes in a dataset. We sometimes do this in order to avoid overfitting the data with a majority class at the expense of other classes whether it's one or multiple. SMOTE has the advantage of not creating duplicate data points, but rather synthetic data points that differ slightly from the original data points. For this reason, SMOTE is considered as a superior oversampling technique. For the prediction modelling part, we used the library of "scikit-learn" in Python. Before feeding the data to the ML algorithms data are standardized by calculating the z-scores using the *StandardScaler* of "scikit-learn"^46^. For the determination of the important predictors, the "Boruta" library was applied^47^.

## Results

### Principal component analysis

The development of a supervised machine learning model that can predict the recovery profile of trauma patients requires labelled data. Since the BIOS data set does not contain patient classifications based on recovery, the first step is to cluster patients based on similarity across the different outcome variables that represent the health condition (unsupervised learning). The topic of similarity-based clustering has been investigated intensively, both from a statistical modeling point of view as well as using Machine/Deep Learning approaches^48^. Preliminary analyses showed that although there is some correlation between the variables measuring recovery, based on clinical expertise it might make sense to separate the recovery variables dealing with physical status from those dealing with psychological function. In the frame of this study, we thus focus on four different cases for the extraction of the clusters: Physical health (with and without pre-trauma scores), psychological health, and general health.

For the case of Physical Health, we implement two cases (i) longitudinal profiles post-injury with four variables namely ‘EQ-5D’, ‘EQ-VAS’, ‘HUI2’ and ‘HUI3’ and (ii) longitudinal profiles including pre-injury data with two variables EQ-5D and EQ-VAS (for which pre-injury values ‘Pre-injury EQ-5D’ and ‘Pre-injury EQ-VAS’ were available). For Psychological Health the variables ‘HDSA’, ‘HDSD’ and ‘IES’ are used. Finally, for the case of General Health, a combination of Physical (no pre-injury values) and Psychological variables is applied (‘EQ-5D’, ‘EQ-VAS’, ‘HUI2’ and ‘HUI3’, ‘HDSA’, ‘HDSD’ and ‘IES’). For General Health, in total forty-two components are present since we have seven variables for six time frames. To investigate how Physical Health variables correlate with Psychological variables PCA was carried out to visualize the correlations between variables and inspect their loading on the principal components.

**Figure 1:**
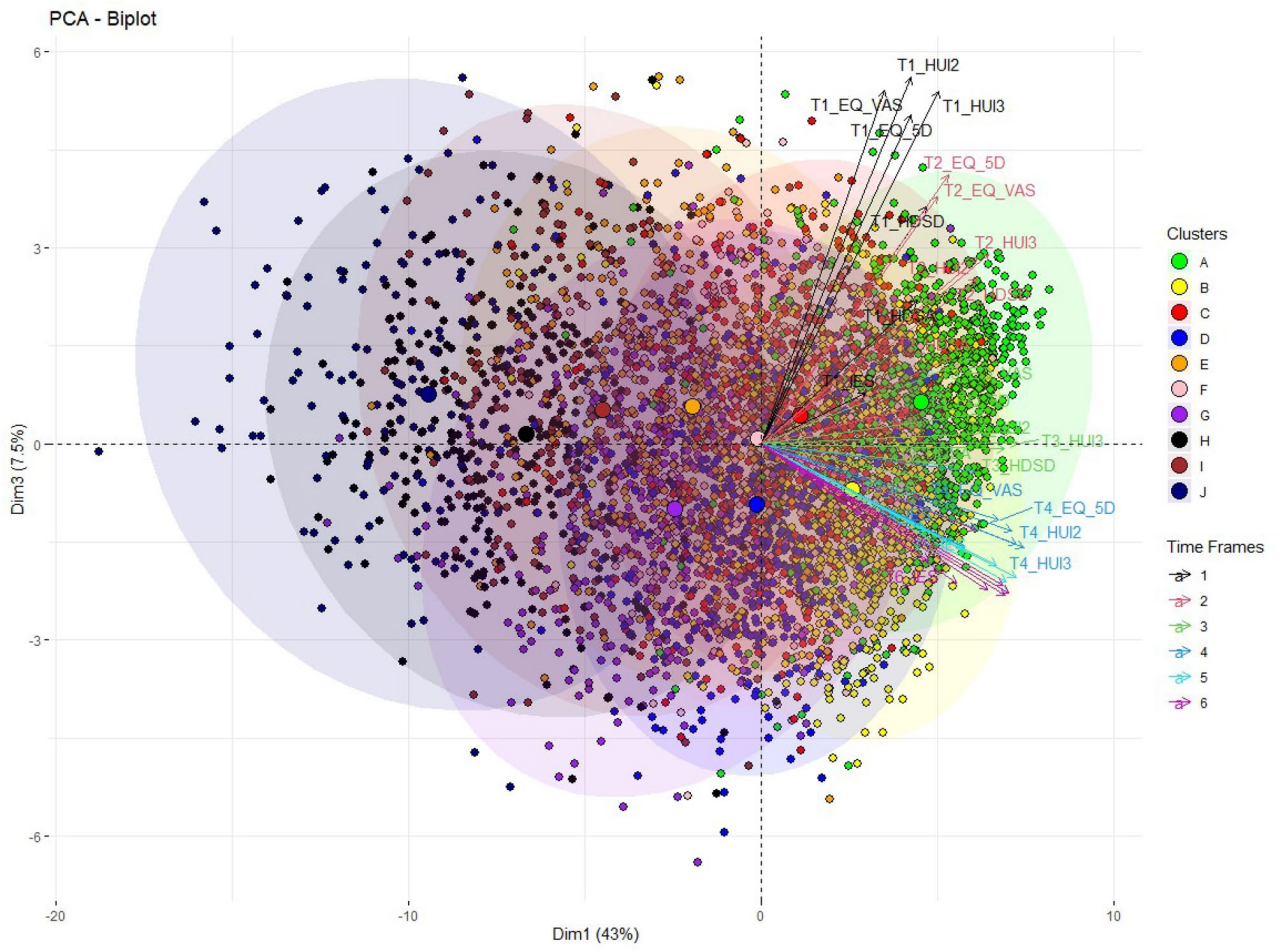
PCA biplot for the set of General Health variables with indication of the ten clusters obtained with kml3d.

The first three components explain more than 60% of the total variance of the forty-two components (see Fig. S4 in the Supplementary information). Additionally, for the first three components, the biggest increase in the cumulative explained variance is observed. For this reason, we extracted the first three components for further analysis. Figure 1 displays the PCA biplot for the first and third component. Different color codes are used to represent the ten patient clusters derived from the set of General Health variables by the kml3d method. The plot shows that the first dimension represents the general health condition of the patients. Positive values on dimension one represent good health of patients two years after trauma while negative values point to poor health. The centers of the clusters move consistently from positive to negative values as we move from the first clusters A, B and C (high initial health and high recovery) to the last clusters J, I and H (low initial health and low recovery) (Fig. 1).

Moreover, we observe that Psychological and Physical Health vectors correlate with each other since they generally point to the same direction for the first dimension. The third dimension represents time as positive values point to variables for the first and the second time frame. As we move to negative values of component three we observe the last three time frames. The correlation of the general health variables is stronger (vectors overlap with each other) as we move from the first to the last time frames. (The second dimension splits the physical from the psychological variables, see Fig. S4 in the Supplementary information).

### Clustering of patients (unsupervised learning)

**Figure 2:**
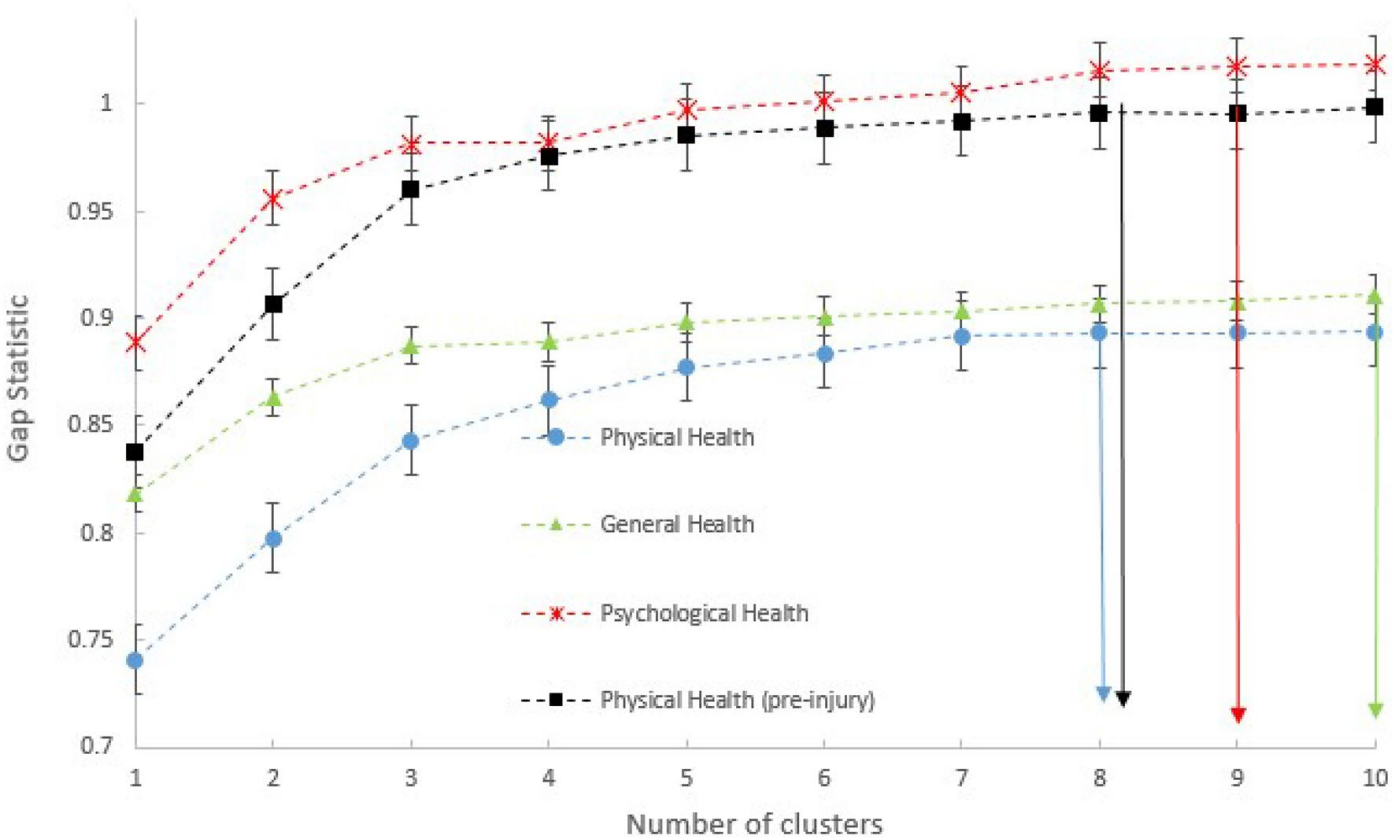
Optimum number of clusters with kml3d for the four different cases of variables and k-means.

For the clusters obtained with kml3d, the optimum number is calculated based on the gap statistic using the "Nbclust" library in R^45^. The gap statistic provides us with the optimal number of clusters per set of variables (Fig. 2). For Physical Health, the optimal number of clusters is eight, for Psychological Health it is nine, for General Health it is ten and finally for Physical Health with pre-injury values it is eight. For the clusters obtained with HDclassif and Deepgmm, a grid search was executed per set of variables in combination with the BIC to determine the optimal number of clusters and setting of the parameters; the results of this are presented in Table 1.

**Table 1:** Tuning parameters for HDclassif and Deepgmm, optimal number of clusters and BIC for the different set of variables (cases).

Case	Method	Algorithm	Initialization	Model	No. of clusters	BIC
Physical health	HDclassif	SEM	k-means	"AKJBKQKDK"	7	− 211,842
Psychological health	HDclassif	EM	k-means	"AKJBKQKDK"	10	− 159,809
General Health	HDclassif	EM	k-means	"AKBKQKDK"	6	− 390,612
Physical health (pre-injury)	HDclassif	EM	k-means	"AKJBKQKDK"	6	− 133,284
Physical health	Deepgmm	–	hclass	–	6	− 254,308
Psychological health	Deepgmm	–	random	–	6	− 180,027
General health	Deepgmm	–	random	–	6	− 469,750
Physical health (pre-injury)	Deepgmm	–	random	–	6	− 137,868

In general, the number of optimum clusters reduced when we apply HDclassif and Deepgmm compared with kml3d. Additionally, clusters obtained with kml3d are generally more balanced (majority baseline is at maximum of 26.15%). On the other hand, unbalanced clusters (except for the case of Physical Health) are obtained when we apply the Deepgmm clustering method (high majority baselines).

### Predicting cluster membership

For predicting the outcome class of the patients, we use the labels generated in the clustering step as the target for prediction in a number of supervised machine learning models. In this following example, we focus the model comparison step for the prediction of the six class labels derived from clustering the set of Physical Health variables including pre-injury values with the HDclassif method. We used Logistic Regression, Random Forest and XGBoost as models with different settings for under- or oversampling and hyperparameters. All models were compared under 5-fold cross validation, and we report the mean f_1_ macro and the 95% CI for accuracy for this example model comparison step in Table 2. We report next to accuracy the f1 macro score since we deal with imbalanced data sets where all the classes are equally important. It is clear that over-sampling has a positive impact on the classification task resulting in higher accuracy and that the Random Forest and XGBoost algorithms outperform logistic regression in this case.

**Table 2:** Example of comparison models for the classification of six clusters obtained for the case of Physical Health (pre-injury) with HDclassif method.

Model	Mean accuracy % (f_1__macro)	95% CI for accuracy	Optimized hyper-parameters
Logistic regression	36.53 (33.33)	[35.60–37.46]	Solver = ‘newton-cg’, C = 10, penalty = ‘*l*_2_’
Logistic regression (under-sampling)	36.11 (34.10)	[34.44–37.79]	Solver = ‘newton-cg’, C = 10^−2^, penalty = ‘*l*_2_’
Logistic regression (smote)	37.88 (35.53)	[36.83–38.94]	Solver = ‘saga’, C = 10^−2^, penalty = ‘*l*_2_’
Logistic regression (over-sampling)	37.20 (35.64)	[35.96–38.45]	Solver = ‘lib-linear’, C = 10^−2^, penalty = ‘*l*_2_’
Random forest	36.98 (35.89)	[34.69–39.27]	Estimators = 200, max depth = 15, min samples split = 5
Random forest (under-sampling)	36.07 (35.12)	[34.24–37.89]	Estimators = 50, max depth = 5, min samples split = 10
Random forest (smote)	54.75 (53. 67)	[52.79–56.70]	Estimators = 500, max depth = 50, min samples split = 2
Random forest (over-sampling)	69.12 (68.71)	[67.81–70.44]	Estimators = 500, max depth = 50, min samples split = 2
XGBClassifier (over-sampling)	68.52 (67.78)	[67.37–69.67]	Estimators = 500, max depth = 10
XGBClassifier (smote)	57.14 (56.23)	[55.95–58.34]	Estimators = 500, max depth = 20

For this reason, Random Forest with over-sampling is the algorithm that we used for the prediction of the classes derived from all clustering attempts using the three clustering methods (Table 3). The best classification results are observed for the clusters obtained with the Deepgmm method. However, the majority baselines for these cluster solutions are high (from 61.02 to 84.70%) meaning that clusters are highly unbalanced. A more detailed methodology for the evaluation of the clusters (clinical sensibleness) based on medical expertise is described in the next section.

**Table 3:** Summary of classification results and quality assessment for clusters obtained from longitudinal data.

Case	Method	Optimum Nr of clusters	Majority baseline	Accuracy (f_1__macro) RF over-sampling	95% CI for accuracy	Clinical sensibleness
Physical Health	kml3d	8	16.48	51.89 (50.03)	[50.84–52.94]	+++
Psychological Health	kml3d	9	26.15	83.12 (82.63)	[82.36–83.87]	++
General Health	kml3d	10	17.07	68.26 (68.02)	[67.66–68.85]	+++
Physical Health (pre-injury)	kml3d	8	18.08	61.56 (60.74)	[60.32–62.81]	++
Physical Health	HDclassif	7	24.49	69.52 (68.61)	[68.00–71.05]	+++
Psychological Health	HDclassif	10	19.12	70.24 (69.75)	[68.78–71.70]	+
**General Health**	**HDclassif**	**6**	**30.03**	**73.96 (72.59)**	**[72.89–75.03]**	**+++**
Physical Health (pre-injury)	HDclassif	6	26.07	69.12 (68.64)	[67.81–70.44]	+++
**Physical Health**	**Deepgmm**	**6**	**45.32**	**91.30 (90.85)**	**[90.50–92.11]**	**+++**
Psychological Health	Deepgmm	6	84.70	99.96 (98.67)	[99.94–99.98]	+
General Health	Deepgmm	6	62.13	98.20 (97.87)	[97.87–98.54]	++
Physical Health (pre-injury)	Deepgmm	6	61.02	94.78 (93.55)	[94.37–95.18]	+

Best models based on classification metrics and clinical sensibleness are in bold.

In order to get a thorough understanding about the prediction, a technique called Boruta is applied to the prediction models^47^. Boruta is a feature selection algorithm, implemented as a wrapper algorithm around Random Forest. In Table 4, the prediction accuracy is presented both with all (26) predictors and only with the important predictors extracted with Boruta for the case of General Health. For kml3d and HDclassif, the same seven predictors are highlighted as important. For the case of Deepgmm, the same predictors are noted as important predictors excluding ‘BMI’ and including predictors such as ‘Category accident’, ‘Education level’, ‘Traumatic brain injury’, ‘Gender’ and ‘Pre-injury cognition’. As can be seen, applying Boruta feature selection did not impair accuracies, leading to simpler models that did not compromise on classification accuracy.

**Table 4:** Significant predictors and accuracy for the case of General Health and different clustering techniques before and after Boruta.

Method	Nr clusters	Accuracy (f_1__macro)	Important Predictors	Accuracy (f_1__macro) with important predictors
kml3d	10	68.26 (68.02)	‘Age’, ‘Injury severity score’,	69.13 (68.23)
			‘Comorbidities’, ‘BMI’, ‘Status score’,	
			‘Pre-injury EQ-VAS’, ‘Frailty’, ‘Admission days in hospital’	
HDclassif	6	73.96 (72.59)	‘Age’, ‘Injury severity score’,	73.82 (72.43)
			‘Comorbidities’, ‘BMI’, ‘Status score’,	
			‘Pre-injury EQ-VAS’, ‘Frailty’, ‘Admission days in hospital’	
Deepgmm	6	98.20 (97.87)	‘Age’, ‘Category accident’, ‘Admission days in hospital’,	98.26 (97.92)
			‘Injury severity score’, ‘Education level’, ‘Comorbidities’,	
			‘Status score’, ‘Pre-injury EQ-VAS’, ‘Frailty’,	
			‘Traumatic brain injury’, ‘Gender’, ‘Pre-injury cognition’	

### Cluster quality evaluation

In the previous section, models with high accuracy were developed for the classification of patients. Specifically, clusters derived from Deepgmm are predicted with high accuracy applying Random Forest and over-sampling. Since the obtained clusters cannot be directly evaluated in terms of representing observable ground-truth classes, the strategy to arrive at sensible and functional models is to combine several quality indicators based on statistical criteria, machine learning metrics, and clusters quality assessment based on medical expertise (clinical sensibleness) in relation to known risk factors for recovery. An example of the applied clusters quality assessment is presented in this section for the clusters obtained with three different methods. For illustration purposes we selected three cases which represent highly, medium and poor sensible clustering (Table 5).

**Table 5:** Descriptive statistics for clusters obtained with different methods and that have been evaluated as Highly (+++), Medium (+ +) and Poorly ( +) sensible.

Case/method	Cluster	Age		Frailty		Comorbidities		Severity score		Admission days in hospital		Gender		Hip fracture	
		Mean	S.E	Mean	S.E	Mean	S.E	Mean	S.E	Mean	S.E	Male %	Female %	No %	Yes %
General Health	1	58.52	0.69	0.63	0.07	0.60	0.04	5.76	0.19	3.91	0.17	65.47	34.53	83.30	16.70
HDclassif	2	61.78	0.48	1.95	0.12	0.88	0.03	6.32	0.13	5.13	0.12	51.98	48.02	78.80	21.20
Highly sensible (+++)	3	64.48	0.64	2.79	0.16	1.08	0.04	6.33	0.16	5.91	0.19	52.73	47.27	77.00	23.00
	4	65.91	0.57	4.86	0.19	1.44	0.04	7.01	0.16	7.50	0.20	39.70	60.30	72.00	28.00
	5	73.89	0.81	5.72	0.27	1.87	0.07	7.02	0.22	8.17	0.40	35.73	64.27	61.40	38.60
	6	74.72	0.93	7.96	0.28	2.04	0.08	7.76	0.28	9.65	0.54	30.95	69.05	54.80	45.20
Physical Health (pre-injury)	A	63.82	0.61	1.89	0.11	0.97	0.04	6.06	0.15	4.83	0.14	49.49	50.51	78.14	21.86
kml3d	B	58.42	0.63	1.06	0.09	0.54	0.03	5.53	0.16	3.93	0.15	66.21	33.79	86.01	13.99
Medium sensible (++)	C	58.06	0.68	1.46	0.15	0.69	0.03	6.72	0.19	5.31	0.17	57.11	42.89	79.77	20.23
	D	69.27	0.66	3.53	0.18	1.58	0.05	6.51	0.18	6.84	0.22	41.43	58.57	69.47	30.53
	E	63.22	0.74	2.81	0.21	1.19	0.04	7.09	0.22	7.30	0.27	43.37	56.63	76.91	23.09
	F	72.78	0.95	6.02	0.28	1.84	0.07	7.72	0.28	10.00	0.49	27.78	72.22	59.34	40.66
	G	73.64	0.84	7.09	0.24	2.05	0.08	7.31	0.28	8.27	0.37	34.96	65.04	62.18	37.82
	H	78.45	0.82	9.47	0.22	2.38	0.08	7.70	0.28	9.60	0.53	28.21	71.79	50.32	49.68
Psychological health	1	64.21	1.44	3.38	0.42	1.19	0.09	6.90	0.38	5.95	0.35	52.02	47.98	70.71	29.29
Deepgmm	2	64.22	2.15	3.78	0.77	1.37	0.16	8.59	0.87	8.67	0.79	40.24	59.76	76.52	23.48
Poorly sensible (+)	3	64.79	0.30	5.04	0.48	1.37	0.09	5.98	0.30	6.40	0.47	39.47	60.53	73.68	26.32
	4	64.84	0.66	3.62	0.1	1.17	0.02	6.54	0.07	6.22	0.10	48.50	51.40	71.95	28.05
	5	66.70	1.82	3.84	0.59	1.34	0.12	6.76	0.57	6.05	0.63	40.87	59.13	74.41	25.59
	6	67.53	1.54	4.31	0.54	1.39	0.11	6.93	0.42	7.38	0.61	37.50	62.50	69.37	30.63

For the case of General Health using the HDclassif method the optimal number of clusters is six. In Table 5 the descriptive statistics per cluster are presented. The order of the clusters is defined from the younger to the older patients. As can be seen, there is a trend for the age of the patients to increase across clusters in this highly sensible model (+++). Specifically, for patients who belong to the first cluster (cluster 1) the mean age is around fifty-eight while for patients who belong to the last cluster (cluster 6) the mean age is around seventy-five. Looking at frailty and comorbidities we observe that older patients are characterized by more comorbidities and higher frailty. Moreover, young patients with less frailty are admitted in the hospital for fewer days and their severity score is also lower compared with patients who are older with more frailty. Additionally, exploring the gender distribution of the clusters we observe that the percentage of females increases as we move from the first to the last clusters. Looking at hip fracture injuries, the clusters quality assessment reveals that the last clusters contain a higher percentage of patients who suffer from this known risk factor for poor recovery. The medium and low clinically sensible models do not recapitulate these demographic risk-factor differences as clearly across clusters.

**Figure 3:**
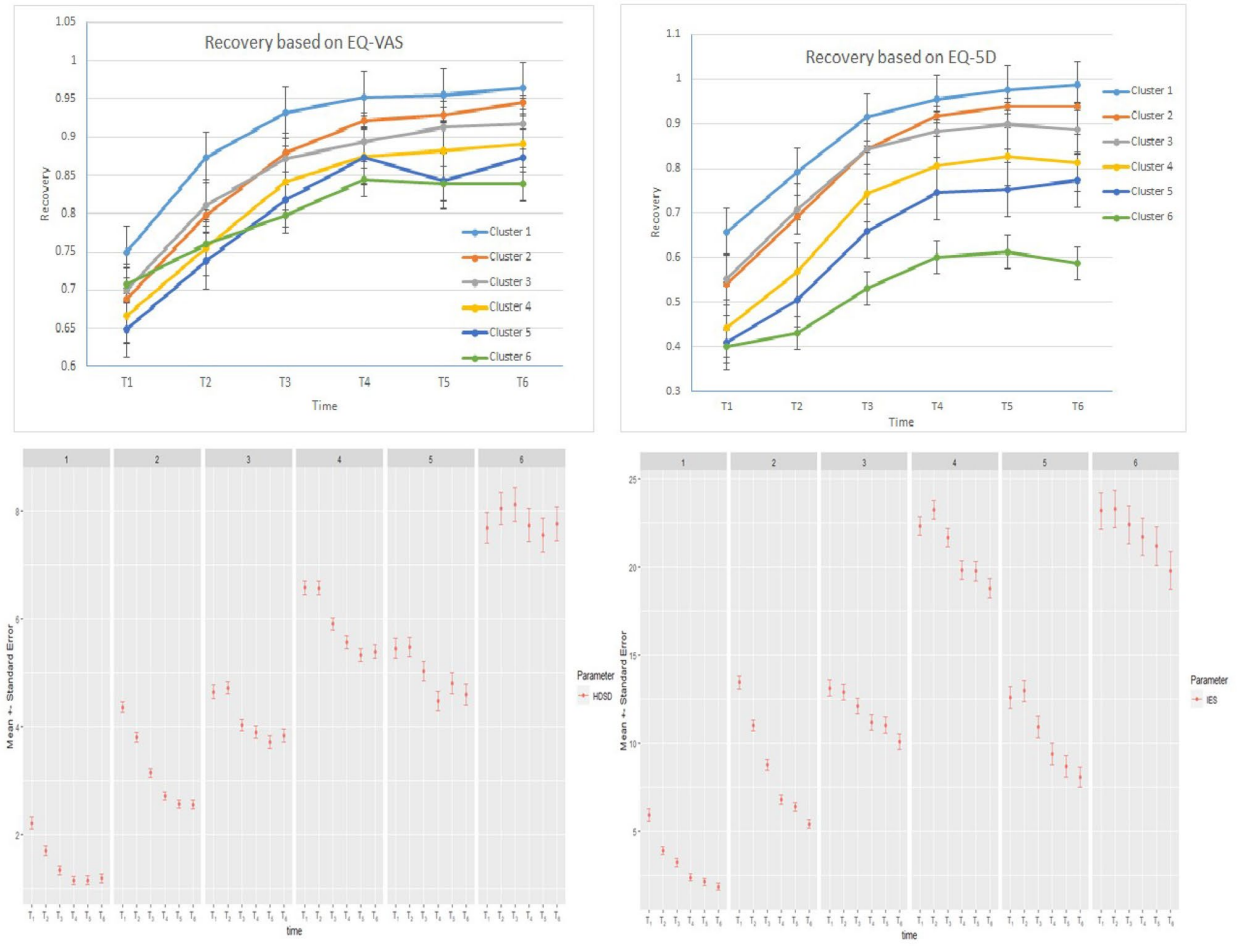
The two graphs at the top present recovery based on EQ-VAS and EQ-5D for the case of General Health with HDclassif. The two graphs at the bottom depict psychological condition (high values indicate high stress and anxiety) of various clusters after the injury.

Recovery of the patients is measured based on various parameters. For two parameters, namely ‘EQ-5D’ and ‘EQ-VAS’, we also have pre-injury estimated baseline values. These variables describe the self-reported physical condition of the patients before their injury. These values can thus be used as a baseline for the analysis of patient recovery (Top two graphs in Fig. 3). As can be seen from the two graphs, EQ-5D and EQ-VAS show a dip from baseline (set at 100%) and show recovery over time. Patients who belong to the first clusters (1, 2) recover almost completely while for patients of the last clusters (5, 6) recovery is about 60–84% depending on the variable.

Psychological condition is also relevant for the recovery of the patients and is plotted in the bottom graphs of Fig. 3. Psychological condition is measured with three parameters namely ‘HDSA’ (Anxiety), ‘HDSD’ (Depression) and ‘IES’. The same trend over time after the accident can be observed for these parameters. More particular, patients who belong to the first clusters (high recovery) appear to have low levels of depression and anxiety. For the first three clusters (1, 2 and 3) the level of stress decreases over time. On the other hand, for clusters 4, 5, and 6 the level of stress and anxiety remains high for a month and then start decreasing.

According to medical experience, the clusters obtained for General Health Case using the HDclassif method meet the expectations and agree with the prototypical cases observed at the hospital. Especially, the group of old females with high frailty and with a hip fracture is a characteristic group observed at the hospital and typically has low recovery. On the other hand, younger male patients with less comorbidities, low severity score and less days admitted to the hospital recover completely and appear to have low levels of stress and anxiety.

For the selection of a rational and functional model that makes clinical sense, we thus implemented a cluster quality assessment as described in the previous paragraphs for each cluster model case. As a reference we use the case of General Health with HDclassif method (highly sensible). The results are presented in Table 3. Based on the clusters quality assessment each case is categorized on clinical sensibleness either as Poorly sensible (+) or as Medium sensible (++) or as Highly sensible (+++). Highly sensible clusters are those cases where the clusters quality assessment reveals discrete clusters with the same trends and characteristics as the reference case (General Health with HDclassif method) matching clinical experience. On the contrary, when we have clusters that are not discrete or without the characteristics of the reference group then the model is categorized as less adequate. This is the case for example for the clusters obtained for Psychological Health with Deepgmm (Table 5). Descriptive statistics of the clusters obtained for this case reveal that clusters are not discrete and do not follow the characteristic trends for frailty, comorbidities, severity score or days admitted in the hospital. Gender and hip fracture do not follow the trend of the reference case.

Performing clusters quality assessment together with medical experts, we discovered that there are cases where clusters partly match with the clusters of the reference case. In this case not all the clusters are discrete. There are clusters which appear similar properties. However, some of the trends of the clusters match with the trends of the reference group. In Table 5 an example of medium sensible case is presented for the case of Physical Health (pre-injury) and the clustering technique of kml3d. For this case although we observe trends between the clusters for the different variables, there are clusters such as (B, C) and (A, E) who are not discrete and do not follow the general trend of the reference clusters More particular, even though cluster C contains patients with slightly lower Age than cluster B, the mean values of ‘Frailty’ and ‘Comorbidities’ are higher.

A supplementary method to quantify the separability of the obtained clusters is to execute a MANOVA. More particular, non-parametric MANOVA (using the function *adonis* from library "vegan"^49^) is executed for the clusters of all cases on the variables of ‘Age’, ‘Frailty’, ‘Comorbidities’, ‘Injury severity score’, ‘Pre-injury EQ-5D’, and ‘*T*_6_-EQ-5D’. We decided to execute a non-parametric MANOVA since the assumptions for running MANOVA (homogeneity of the variances and normality within the groups) were not met for our data. Assumptions are examined using the function *assumptions manova*. The non-parametric MANOVA revealed that there was a strong relation between the value of the F statistic and the sensibility of the clusters. More precisely, F values between 79.71 and 101.45 (separable clusters, very low p-values) are obtained for the highly sensible models. For medium sensible clusters F is between 5.99 and 8.32 while for inadequate clusters F is between 1.21 and 2.98. For the non-sensible clusters, the difference between clusters is not significant, showing p-values larger than the chosen threshold of 5%. It is remarkable that for the case of Physical Health with Deepgmm method, non-parametric MANOVA reveals that there is a statistically significant difference between the obtained clusters, F(35, 3880) = 101.45, p < 10^−3^. On the contrary, for the case of Psychological Health with Deepgmm, non-parametric MANOVA indicates that the separability of the clusters is not statistically significant F(35, 3880) = 1.21, p = 0.30.

Further evaluation of the models is performed by using a graphical method: plotting the t-distributed stochastic neighbor embedding (t-SNE) graphs. In the Supplementary information the t-SNE graphs of two extreme cases, namely General Health with kml3d and 10 clusters with high clinical sensibleness and Psychological Health with Deepgmm with 6 clusters with low clinical sensibleness, are presented (see Fig. S5 and Fig. S6 in the Supplementary information). In the case of General Health with kml3d, t-SNE visualisation shows discrete clusters in the two-dimensional space. On the contrary, for Psychological Health with Deepgmm, high interference between the groups is observed.

From Table 3, we observe that for General Health, the best model is achieved with the HDclassif method. The accuracy of this model is almost 74% while the clusters quality assessment indicates that the obtained clusters are sensible. For the case of Physical Health, the best model with high accuracy (91.30%) and sensible clusters is derived using the Deepgmm method. Cluster quality assessment of clusters obtained with the HDclassif method for Physical Health with pre-injury measurements reveals that clusters are highly sensible, however, accuracy is much lower (at 69.12%) compared with Deepgmm. Another observation has to do with the case of Psychological Health. Applying variables which are related only to the psychological condition of the patients do not lead to sensible (+++) clusters for any method, suggesting that these outcome measures are not related to traditional risk factors for physical recovery, but capture a different dimension.

## Discussion

Here, we have developed a robust multi-step approach for selecting and evaluating machine learning methods to predict longitudinal recovery profiles of patients after trauma. Longitudinal clusters were created through three different methods and evaluated on cluster quality, clinical sensibleness and accuracy of prediction when the cluster labels were used as targets for supervised learning with Random Forest. The models that combine high clinical sensibleness with good prediction performance forecasting the clustered outcomes based on patient demographic data have the potential to be used as a reliable and intuitive tool in the shared decision making processes between clinician and patient. In such discussions, the projected recovery and potential interventions in the case of poor predicted outcome could be presented.

Principal Component Analysis indicated that variables that represent the physical health of the patients correlated with variables that represent the psychological condition. However, the clustering of the patients based on only psychological variables did not result in clusters characterized by high clinical sensibleness. In contrast, applying variables that represent only physical health or general health (physical plus psychological) result in clusters with high clinical sensibleness. This could be due to the fact that psychological variables are not as highly correlated as physical health for the for the different time frames and dimension 1 (which represents health condition) as shown in by the Principal Component Analysis (see the plot with Dimension 1 and 2 in the Supplementary information Fig. S4). Another explanation is related to the nature of the BIOS dataset which gives emphasis mainly on the physical health recovery (4 measures) of the patients and less on psychological health (3 measures).

The results of Random Forest with and without feature selection showed that highly accurate prediction does not require a high number of predictors suggesting that only few predictors are important and the rest introduce noise to the model. Exploring the important predictors, we found that all of them are relevant to the health of patients. More particularly, predictors such as ‘Age’, ‘Comorbidities’, ‘BMI’, ‘Pre-injury EQ-5D’, ‘Pre-injury EQ-VAS’ and ‘Frailty’ relate directly to health. Although quality assessment of the clusters suggest that BMI is not a discrete property between the clusters, Boruta revealts that BMI is a significant property for classification, in accordance with previous research^50,51^. According to medical experts, BMI does correlate with the health of a patient, with extreme BMI values as an indication for poor health. The ‘Injury severity Score’ (ISS) is a measure to describe the overall severity of the injury. Although the ISS is developed as a predictor for in-hospital mortality, it also predicts morbidity after injury^52^. Thus high severity indicates a serious accident with low possibilities to recover completely compared to an accident of low severity. The same trend is valid for ‘Admission days in hospital’. Higher values for ‘Admission days in hospital’ indicate a more serious injury or indicates the presence of complications, which affect recovery after injury^53^. All the above predictors have been highlighted as significant for the physical outcome in previous studies using multi-variable regression analysis^13,34^.

Our methodology of generating clusters of patients ad-hoc by the usage of unsupervised learning techniques and subsequently making predictions has provided excellent classification results. This ad-hoc method has not been implemented in previous literature as far as we know. The results show that besides the usage of measures such as BIC and the gap statistic to find the best clustering method, medical expertise is also essential for the evaluation of the machine learning models. During the evaluation of the models with medical experts a quality assessment based the descriptive statistics of the clusters was performed to evaluate how well the clusters aligned with clinical observations. For some cases high classification accuracy was achieved. However, by exploring the clusters with descriptive statistics we conclude that clusters are not discrete and do not agree with the expectations of medical experts and known risk factors.

Considering the complexity of making predictions in a multi-class problem, the resultant analysis from this research work is promising. In our case, the traditional machine learning methods like Random Forests are performing excellent. However, most of previous studies used neural networks^54^. The implementation of deep neural networks for the classification part is an option for future work. However, the small number of patients in the BIOS study do not promise that more advanced deep learning method will result in better classification metrics. Additionally, more advanced clustering techniques can be applied to separate patients in different categories. An example of these methods is a variational deep embedding with recurrence (VaDER). VaDER relies on a Gaussian mixture variational auto-encoder framework, which is further extended to (i) model multivariate time series and (ii) directly deal with missing values^55^. The implementation of multiple hidden layers for Deepgmm method is also an option for the extraction of clusters^44^. Optimization of the Deepgmm models for the number of hidden layers and the number of components per layer together with machine learning classification metrics will form the topic of further research.

To conclude, it should be noted that although recovery outcome prediction can be improved with machine learning techniques, a model should not replace expert opinion and contextualization. Instead, accurate ML models for the assessment of recovery after trauma could assist with communication in the consulting room between the medical professional and patient, leading to timely interventions that may reduce physical and psychological complaints and potentially reduce societal costs^56^. ML models could be deployed in clinical practice, operating on the electronic patients files and updated with new incoming data. Furthermore, the models go beyond physical recovery and also incorporate other aspects of health; psychological outcome, self-care and daily activities, which highlight the need for a holistic approach concerning rehabilitation after trauma.

## Supplementary Information

Supplementary Information.

## Data Availability

The datasets generated and/or analysed during the current study are not publicly available due to high risk of re-identification but are available from the corresponding author on reasonable request.
