# Supplementary material for: Clustering of trauma patients based on longitudinal data and the application of machine learning to predict recovery
Source: Sci Rep. 2022 Oct 10;12:16990. doi: 10.1038/s41598-022-21390-2 (PMC9550811; doi:10.1038/s41598-022-21390-2)
Supplement: Supplementary file 1 — Supplementary Information. [file 41598_2022_21390_MOESM1_ESM.pdf]

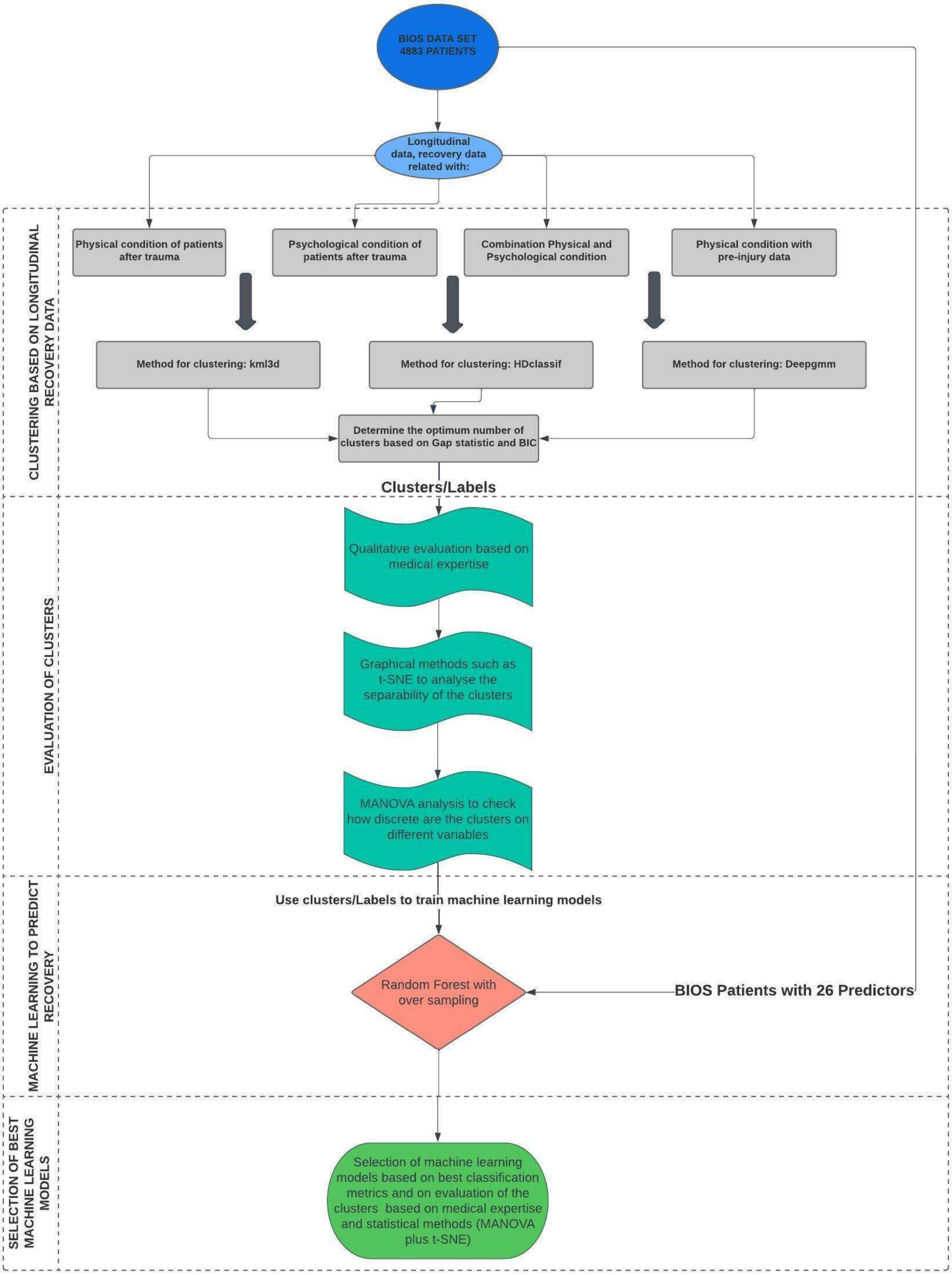

Figure S1. Schematic overview of the study.

Variables (used for)	Numerical or Categorical	Range	Related info
EQ-5D (clustering)	Numerical	Range 0-1	The EQ-5D™ measures health status in five dimensions: mobility, self-care, usual activities, pain/discomfort and anxiety/depression. Each dimension has three possible levels: no problems, moderate problems or severe problems. A utility score (EQ-5D™ utility) was calculated, ranging from 0 representing death to 1 for full health.
EQ-VAS (clustering)	Numerical	Scale 0-100	Health status. The EQ-VAS ranged from 0 at the bottom (worst possible health state) to 100 at the top (best possible health state).
HUI2 (clustering)	Numerical	Range 0-1	HUI is used to measure general health status. It consists of 15 questions, divided into seven HUI Mark 2 (HUI 2) questions and eight HUI Mark 3 (HUI 3) questions. A utility score was calculated using an algorithm to quantify health status, where dead scores 0 and perfect health scores 1.
HUI3 (clustering)	Numerical	Range 0-1	
HDSA (clustering)	Numerical	Range 0-10	Record symptoms of anxiety. High values indicate high levels of anxiety.
HDSD (clustering)	Numerical	Range 0-10	Record symptoms of depression. High levels indicate high levels of depression.
IES (clustering)	Numerical	Range 0-25	The Impact of Events Scale (IES) was used to measure Post Traumatic Stress Symptoms.
Pre-injury EQ-5D (clustering/prediction)	Numerical	Range 0-1	Patients were asked about their health status before accident by filling out the EQ5D-3L.
Pre-injury EQ-VAS (clustering/prediction)	Numerical	Scale 0-100	Patients were asked about their health status before accident by filling out the EQ-VAS.
Age (prediction)	Numerical		Age in years of patient at arrival emergency department.
Category accident (prediction)	Categorical	1-7	1: Violence<br>2: traffic<br>3: work<br>4: private<br>5: sport<br>6: self-Mutilation/Suicide<br>7: other

Admission days in hospital (prediction)	Numerical		Length of hospital stay in days.
Injury severity score (prediction)	Numerical	Range 5-10	(ISS) Injury Severity Score (ranging from 5: minor injury to 10: fatal injury).
Frailty (prediction)	Numerical	Range 0-10	Frailty was measured at one week or one month after injury with the Groningen Frailty Index and refers to weakness before the accident. Low values indicate good health while high values is an indication of bad health.
Educational level (prediction)	Categorical	Low,<br>Middle,<br>High	Educational level was categorised in three levels as the highest completed degree, diploma of education: low (primary education, preparatory secondary vocational education or without diploma), middle (university preparatory education, senior general secondary education or senior secondary vocational education and training), and high (academic degree or university of applied science).
Comorbidities (prediction)	Numerical		Number of comorbidities.
BMI (prediction)	Numerical		Body Mass Index.
Status score (prediction)	Numerical	the score ranges from -6.75 to 3.06	Status score was based on home postcode. All postcodes in the NL correspond to a specific status score, based on the level of education, income and percentage unemployment in the neighborhood; the score ranges from -6.75 to 3.06, with a lower value indicating low status and vice versa. In 2014, the mean status score in the NL was 0.28.
Traumatic brain injury (prediction)	Categorical	Yes/No	Traumatic brain injury (TBI) (Head injury).
ICU (prediction)	Categorical	Yes/No	Admission to the intensive care (yes/no).
Type Comorbidity (prediction)	Categorical	1-4	Comorbidity before admission to the hospital.<br>1: Frailty healthy patient<br>2: patient with mild systemic disease, controlled well.<br>3: patient with severe systemic disease, limits normal activities.<br>4: patient with very severe systemic disease, which is a constant threat to life.
Gender (prediction)	Categorical	Male/Female	

Pelvic injury (prediction)	Categorical	Yes/No	
Hip fracture (prediction)	Categorical	Yes/No	
Shoulder and upper arm injury (prediction)	Categorical	Yes/No	
Radius, ulna or hand fracture (prediction)	Categorical	Yes/No	Under arm or hand fracture.
Facial injury (prediction)	Categorical	Yes/No	
Thoracic injury (prediction)	Categorical	Yes/No	Injury of lungs or heart.
Rib fracture (prediction)	Categorical	Yes/No	
Spinal cord injury (prediction)	Categorical	Yes/No	
Stable vertebral fracture or disc injury (prediction)	Categorical	Yes/No	
Abdominal injury (prediction)	Categorical	Yes/No	
Pre-injury cognition (prediction)	Categorical	No problems/Mild/Severe	Patients were asked about their cognition status before accident.

Table S2. An overview of the variables used for clustering and prediction.

**Classifier**	**Hyper-parameters**		
Logistic regression	Solver (‘newton-cg’, ‘liblinear’, ‘sag’ and ‘saga’)	C (10^{-4}, 10^{-2}, 1, 10, 50)	Penalty (‘l_1’, ‘l_2’, ‘elastic-net’)
Random Forest	Number of estimators (5, 10, 50, 100, 200, 500)	Depth (2, 5, 10, 15, 20, 50, 100)	Minimum sample size per split (2, 3, 5, 10)
XGBoost	Maximum depth (2, 3, 4, 6, 10, 20, 100)	Number of estimators (10, 50, 100, 200, 500)	

Table S3. An overview of the hyper-parameters tuned during training.

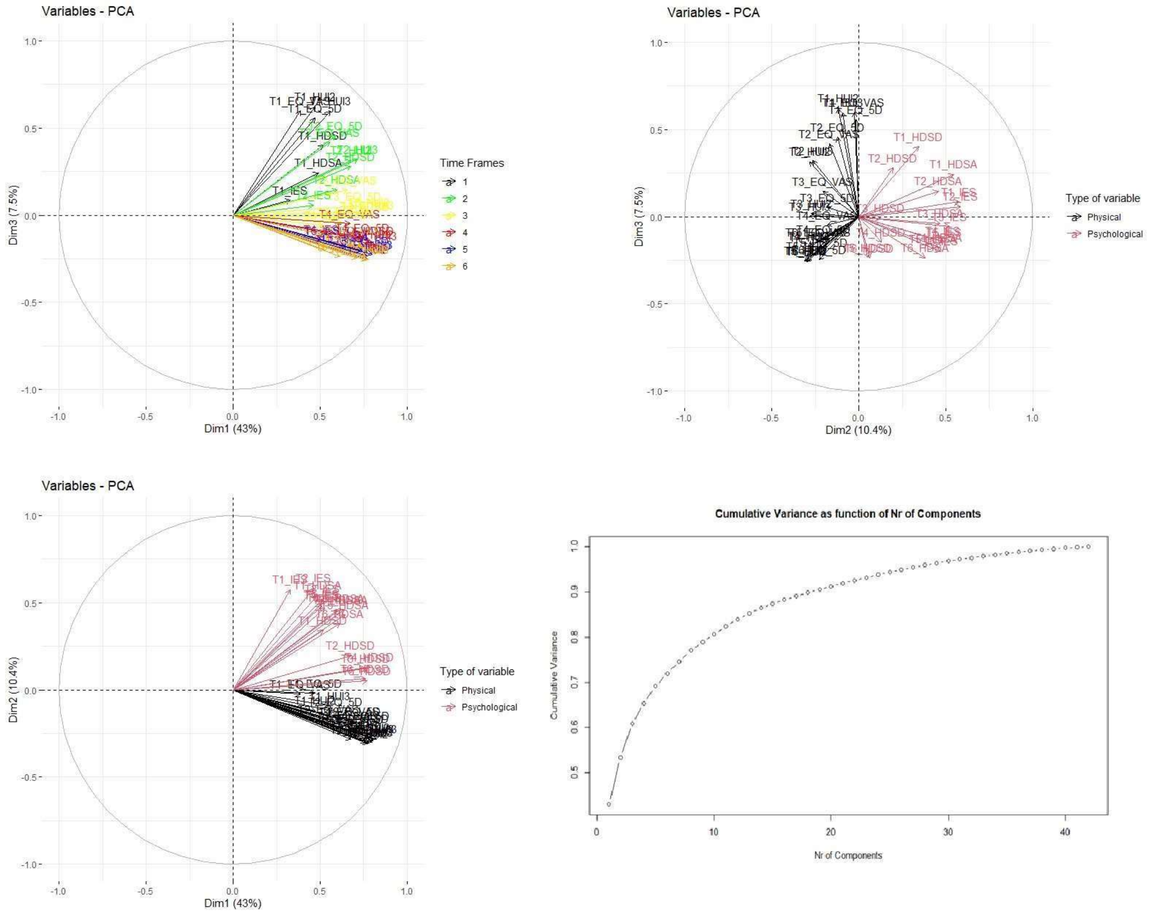

Figure S4. PCA graphs for Psychological and Physical Health variables for different PCA components. Dimension 2 splits the variables in Physical and Psychological while Dimension 3 represents time.

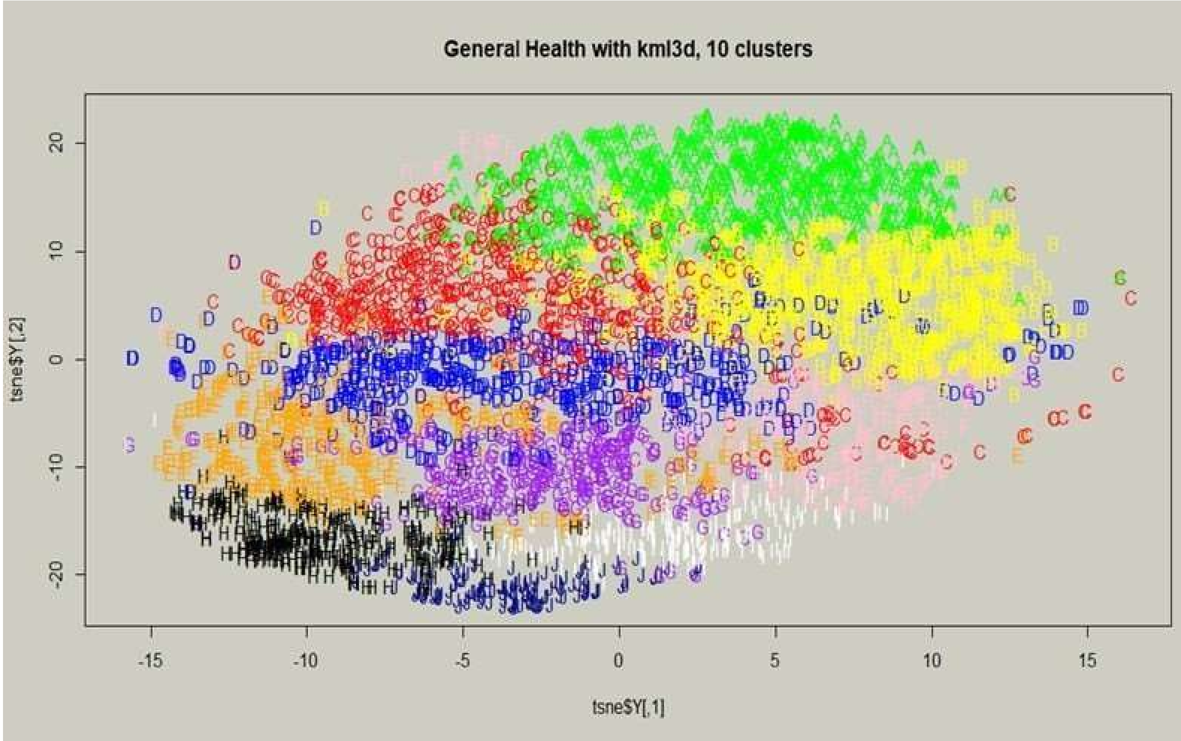

Figure S5. t-distributed stochastic neighbor embedding (t-SNE) graph for the case of General Health with kml3d and 10clusters.

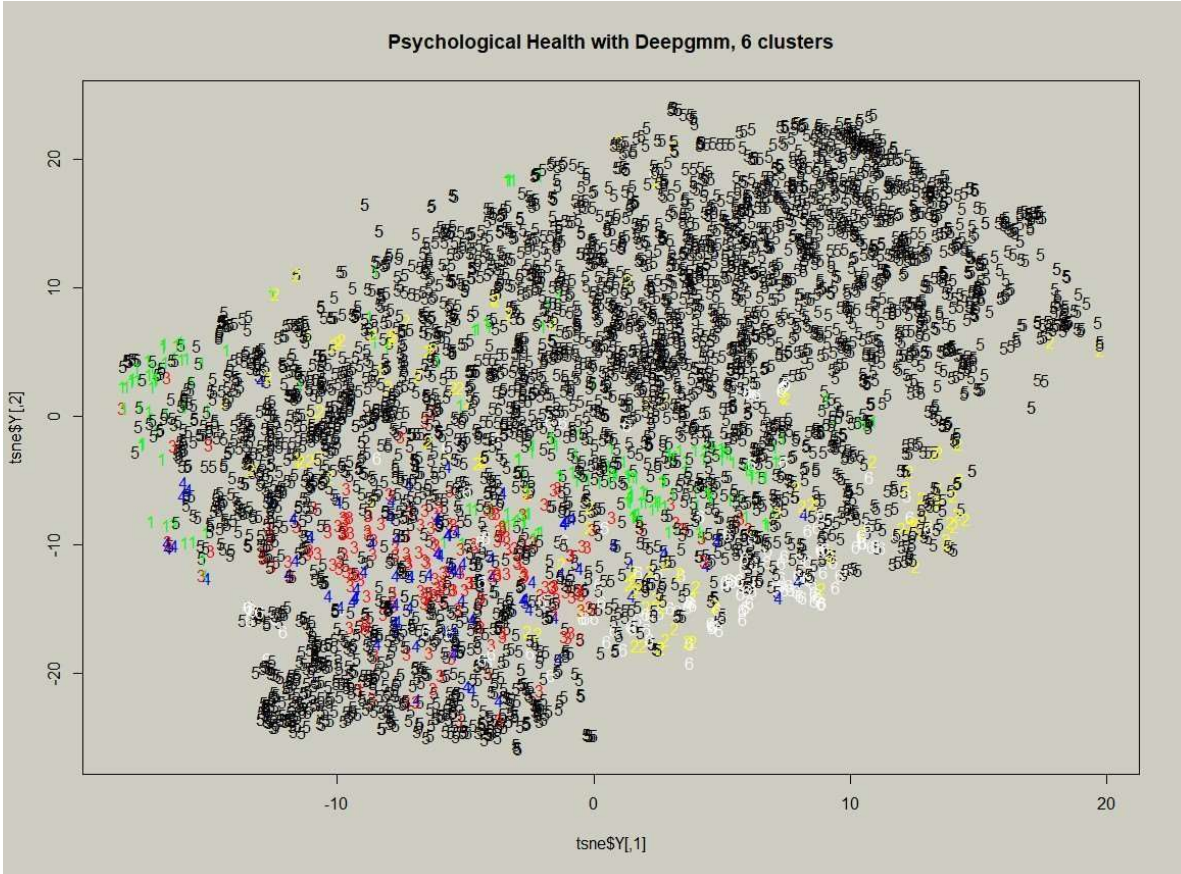

Figure S6. t-SNE graph for the case of Psychological Health with Deepgmm and 6 clusters.
